# The complete chloroplast genome of Tomentosa cherry *Prunus tomentosa* (Prunoideae, Rosaceae)

**DOI:** 10.1080/23802359.2018.1476068

**Published:** 2018-06-11

**Authors:** Tao Chen, Yan Wang, Lei Wang, Qing Chen, Jing Zhang, Hao-Ru Tang, Xiao-Rong Wang

**Affiliations:** a College of Horticulture, Sichuan Agricultural University, Chengdu, China;; b Institute of Pomology and Olericulture, Sichuan Agricultural University, Chengdu, China

**Keywords:** Chloroplast genome, Illumina sequencing, *Prunus tomentosa*, phylogenetic analysis

## Abstract

*Prunus tomentosa* belongs to subgenus *Lithocerasus* in family Rosaceae. It is native to northern China and has become a staple back yard garden plant in Russia and much of Eastern Europe. In this study, *de novo* assembly with low coverage whole genome sequencing facilitated to generate the complete chloroplast (cp) genome of *P. tomentosa*. The genome size is 158,356 bp in length. It exhibited a typical quadripartite structure comprising a large single copy region (LSC, 86,630 bp), a small single copy region (SSC, 19,010 bp) and a pair of inverted repeat regions (IRs, 26,358 bp each). A total of 115 genes were predicted including 82 protein-coding genes, 29 tRNA genes, and four rRNA genes. Phylogenetic analysis indicated that *P. tomentosa* is most closely related to *P. mume* suggesting the phylogenetic relationship between *P. tomentosa* and subgenus *prunpphora*.

*Prunus tomentosa* Thunb. (commonly Tomentosa cherry or Nanking Cherry) is an important deciduous fruit tree endowed with highly ornamental and economic values (Yü et al. 1986). It is native to northern China and naturalized in Japan, Russia, and other northern regions of the continent (Hamilton et al. 2016). For centuries, *P. tomentosa* has possessed many valuable traits, such as various peel colour, very cold tolerant, diverse environments adaption (Zhang et al. 2008). It can be used as a gene donor to improve other *Prunus* species. However, such breeding programs require a sufficient understanding of the groups’ phylogeny among different species (Zhang and Gu 2016). The phylogenetic relationship of *P. tomentosa* was always controverted (Shi et al. 2013). In this study, we generated the complete chloroplast (cp) genome sequence of *P. tomentosa*, which could help us verify the phylogenetic relationship between *P. tomentosa* and its relative species.

The plant material was obtained from Changyi, Shandong province, China (35°45.5146′ N, 119°26.4163′ E, altitude 25 m). Total DNA was extracted with a modified CTAB protocol. An Illumina paired-end (PE) library was constructed and sequenced using an Illumina HiSeq 2500 platform (Illumina, San Diego, CA). After quality trimming, a total of 2.35 Gb clean PE reads (Phred scores >20) were assembled using SOAPdenovo (Li et al. 2009). Contigs were ordered and merged with the cp sequence of *Prunus persica* (NC_04697). Further validation was also performed using manual correction. The plastom was annotated by Dual Organellar GenoMe Annotator (DOGMA) (Wyman et al. 2004) (http://dogma.ccbb.utexas.edu/).

Similarly, complete cp genome sequence of other 11 Rosales species (*Pyrus pyrifolia* NC015996 and *Pyrus spinosa* NC023130 as outgroups) were aligned using MAFFT 5 (Katoh et al. 2005). Maximum likelihood (ML) analysis was implemented in RAxML v8.2.4 (Stamatakis 2006). Maximum parsimony (MP) and neighbour-joining (NJ) analysis were performed using MEGA 6.0 (Tamura et al. 2013) (http://www.megasoftware.net/).

The circular genome of *P. tomentosa* is 158,356 bp in size, with overall GC content 36.85%. It comprises a large single copy (LSC) region of 86,630 bp, a small single copy (SSC) region of 19,010 bp and a pair of inverted repeat (IR) of 26,358 bp. A total of 115 unique coding regions were predicted, comprising 82 protein-coding genes, 29 tRNA genes, and four rRNA genes. Among all unique genes, 19 genes contain one intron, two genes (*ycf3* and *clpP*) with two introns, and gene *atpB* has three introns. All the coding regions accounted for 58.35% of the whole genome. The genome sequence was deposited in GenBank with the accession number MF_624726.

Phylogenetic analysis revealed three subgroups in genus *Prunus* (Figure 1). Five species from subgenus *Creasus* formed one group, subgenus *Padus* and *Maddenia* composed of another clade. Interesting, *P. tomentosa* was nested within subgenus *Amygdalus* and *prunpphora* constituted the third group, which was, in turn, a sister to *P. mume*. This result was congruent with previous studies by isozyme (Shi et al. 2013) and other molecular markers (Chin et al. 2010).

**Figure 1.**
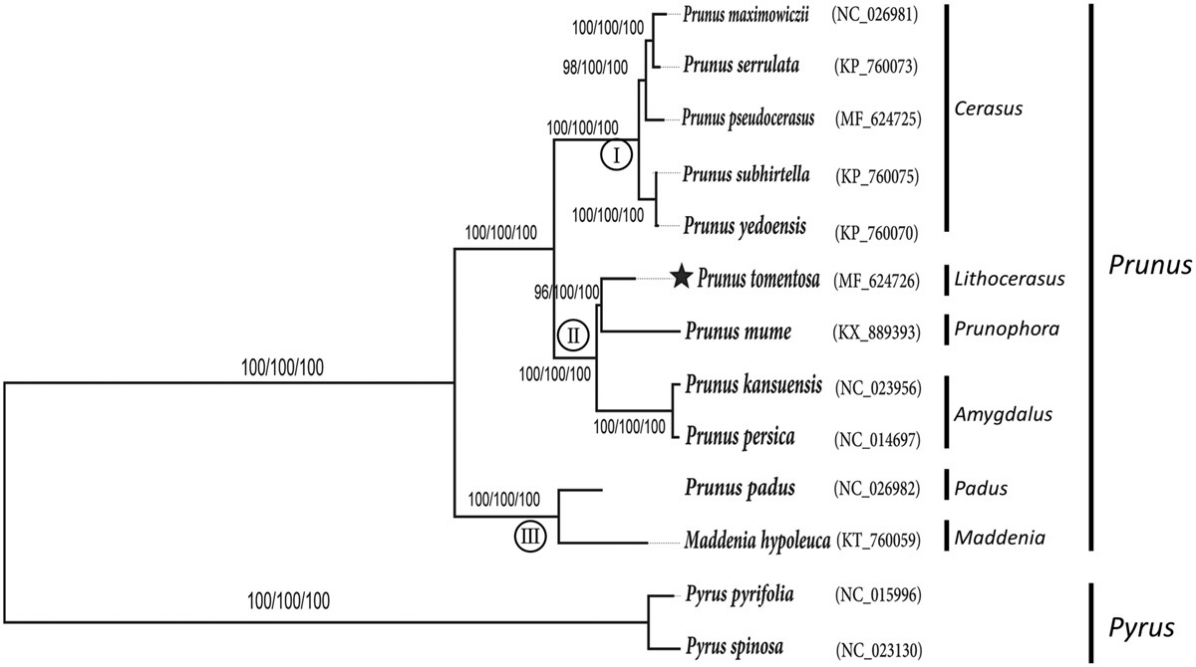
Phylogenetic tree of *P. tomentosa* and other 12 species belonging to the Rosaceae. Tree was inferred from the complete chloroplast genome sequences using the ML method with a GTR model, MP method, and NJ method with a K-2P model. Only the framework of the ML tree was presented. Numbers in the nodes were the bootstrap values from 1000 replicates with an arrangement of ML/MP/NJ methods. Symbol (I, II, III) in the nodes represents three groups in genus *Prunus*.
